# Mushrooms: A Potential Natural Source of Anti-Inflammatory Compounds for Medical Applications

**DOI:** 10.1155/2014/805841

**Published:** 2014-11-23

**Authors:** Elsayed A. Elsayed, Hesham El Enshasy, Mohammad A. M. Wadaan, Ramlan Aziz

**Affiliations:** ^1^Bioproducts Research Chair, Zoology Department, Faculty of Science, King Saud University, Riyadh 11451, Saudi Arabia; ^2^Natural and Microbial Products Department, National Research Centre, Dokki, Cairo 12311, Egypt; ^3^Institute of Bioproduct Development (IBD), Universiti Teknologi Malaysia (UTM), 81130 Skudai, Malaysia; ^4^City of Scientific Research and Technology Application, New Burg Al Arab, Alexandria 21934, Egypt

## Abstract

For centuries, macrofungi have been used as food and medicine in different parts of the world. This is mainly attributed to their nutritional value as a potential source of carbohydrates, proteins, amino acids, and minerals. In addition, they also include many bioactive metabolites which make mushrooms and truffles common components in folk medicine, especially in Africa, the Middle East, China, and Japan. The reported medicinal effects of mushrooms include anti-inflammatory effects, with anti-inflammatory compounds of mushrooms comprising a highly diversified group in terms of their chemical structure. They include polysaccharides, terpenoids, phenolic compounds, and many other low molecular weight molecules. The aims of this review are to report the different types of bioactive metabolites and their relevant producers, as well as the different mechanisms of action of mushroom compounds as potent anti-inflammatory agents.

## 1. Introduction

In the human body, inflammation is considered to be part of the complex biological response to remove injury or harmful stimuli such as pathogens, damaged cells, or irritation. This response leads to many physical symptoms such as fever, pain, and swelling, as a result of many associated changes such as vasodilation, increased vascular permeability, and plasma extravasation. Nowadays, the nonsteroidal anti-inflammatory drugs (NSAIDs) are usually the most commonly administrated drug to reduce inflammation in the body. Many studies, however, have shown that the long-term administration of NSAIDs has the potential for significant side effects on the gastrointestinal tract (GIT). These include numerous harmful effects such as mucosal lesions, bleeding, peptic ulcers, and intestinal perforation [1, 2]. Other studies, meanwhile, have suggested that the side effects of NSAIDs are not limited only to GIT but extend to other serious complications such as acute renal failure, nephrotic syndrome, hypertension, and cardiovascular toxicity [3–5]. Recently, therefore, much effort has been devoted towards the discovery of alternative anti-inflammatory compounds of plant origin as potential natural and safe medicines without the harmful side effects of NSAIDs [6–11]. While a variety of plants have traditionally been used in human medicine, mushrooms also have a long history as important components of folk medicine and have been widely used in the form of aqueous extracts in many African, Middle Eastern, European, Asian, and native Australian cultures for the treatment of different diseases as well as a preventive medicine [12–16].

Mushrooms are a very large and diversified group of macrofungi belonging to Basidiomycetes and Ascomycetes; with a cell cycle including the formation of sexual spores. The fungal spores for these two groups are located in a special structure called the basidium (for Basidiomycetes) or the ascus (for Ascomycetes), and such mushrooms can grow either above the earth (epigeous macrofungi), giving mainly umbrella like structures which include basidiospores, or at depths of 10–20 cm below the soil surface (hypogeous macrofungi or truffles). The latter of these belong mainly to Ascomycetes and usually grow in a symbiotic relationship with a host plant as ectomycorrhizal mushrooms (EMM).

Both types of mushrooms have considerable nutritional value, since they are rich sources of carbohydrates, proteins, free amino acids, and vitamins, as well as different essential minerals and trace elements [17, 18]. They are also rich in many bioactive metabolites of high medicinal value such as lectins, polysaccharides, phenolics and polyphenolics, terpenoids, ergosterols, and volatile organic compounds [19–21]. Mushroom extracts have therefore been used medicinally in immunomodulator [22–25], antitumor/anticancer [26–30], antibacterial and antiviral [31–33], antioxidant [34–37], and antihypoglycaemic [38–40] applications and as active medicines in the prevention of cardiovascular diseases through their action as antiatherosclerotic agents [41]. In addition, many compounds of highly diversified chemical structures with anti-inflammatory activities have been isolated and purified from different types of mushrooms. It has been reported, for example, that water, methanolic, ethanolic, and ethyl acetate extracts of different epigeous and hypogeous mushrooms showed significant decreases in the activities of inflammatory mediators such as nitric oxide (NO), cytokines, and prostaglandins, thus inhibiting some macrophage functions and reducing cell inflammations.

## 2. Inflammation and Cell Molecular Signaling

Inflammation is a complex set of interactions among soluble factors and cells that can arise in any tissue in response to trauma, infections, or postischaemic, toxic, or autoimmune injury [42]. In normal cases, the body's response to inflammation is self-limiting through the downregulation of proinflammatory protein expression, the increased expression of anti-inflammatory proteins, and a reversal in the vascular changes that facilitated the initial immune cell recruitment process [43]. The processes leading to inflammation are usually linked to the activities of the cells involved in the restoration of tissue structure and function. When cells are exposed to immune stimulants, the proinflammatory cells, such as macrophages, monocytes, or other host cells, start to produce many molecular mediators which initiate the inflammation process. Of the various inflammatory biomarkers that are produced, the most well-known are interleukins (IL-1*β*), IL-6; IL-8; tumour necrosis factor (TNF-*α*); nuclear factor-*κ*B (NF-*κ*B), intercellular adhesion molecule-1 (ICAM-1), inducible type cyclooxygenase- (COX-) 2, prostaglandin E2 (PGE2); 5-lipooxygenase (5-LOX); and inducible nitric oxide synthase (iNOS), which leads to the production of reactive nitrogen species such as nitric oxide (NO). Overproduction of these inflammatory mediators leads to different kinds of cell damage. In addition, prolonged inflammation causes many inflammatory diseases such as juvenile idiopathic arthritis (JIA), inflammatory bowel disease (IBD), multiple sclerosis, rheumatoid arthritis, gastritis, bronchitis, and atherosclerosis [44]. A strong link has also been established between long-term inflammation and the development of cancers [45]. In some types of cancer, inflammation occurs before malignant changes in the cells. In other types, oncogenic change induces an inflammatory microenvironment that promotes tumour development [46]. As a result, increased attention is now being focused on efforts to discover bioactive compounds which have the ability to suppress the production of inflammatory mediators. In this context, mushroom metabolites have been employed as potent, natural, and safe anti-inflammatory compounds based on their ability to reduce the production of inflammatory mediators through downregulation of the gene expression of different types of these inflammatory mediators.

## 3. Anti-Inflammatory Compounds of Mushrooms and Modes of Action

Mushrooms are widely used for their high nutritional value as a functional food. Additionally, they have been highly appreciated for their medicinal and therapeutic applications [47, 48]. Edible mushrooms produce a vast diversity of bioactive compounds such as polysaccharides, proteoglucans, terpenoids, phenolic compounds, steroids, and lectins. These compounds have a wide range of therapeutic effects and can act as immune-modulatory, anticarcinogenic, antiviral, antioxidant, and anti-inflammatory agents [16, 20]. The concentration and efficacy of the bioactive compounds are varied and depend on the type of mushroom, substrate applied, cultivation and fruiting conditions, stage of development, age of the fresh mushroom, storage conditions, and processing and cooking procedures [41]. Many of the bioactive compounds found in mushrooms exhibit significant anti-inflammatory properties.  Table 1 lists most of the well-known mushroom species reported in the literature as possessing anti-inflammatory activities, along with their bioactive compounds and the solvent applied for extraction.

### 3.1. Polysaccharides

**Figure 1:**
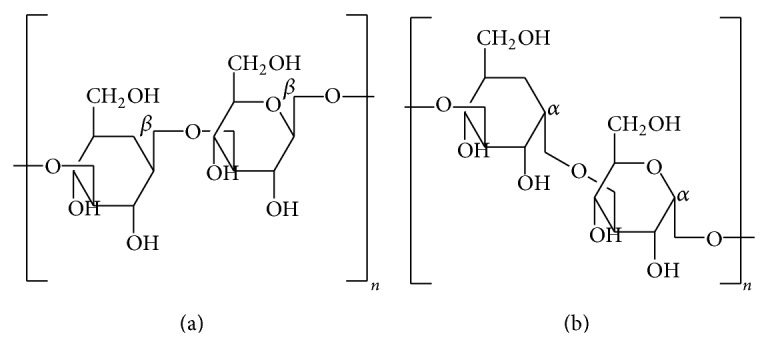
Molecular structure of *β*-(1→3)-D-glucans (a) and *α*-(1→3)-D-glucans (b).

Polysaccharides represent the major class of bioactive compounds found in mushrooms, and have been reported in most of the edible types. Bernardshaw et al. [49] investigated the effect of an aqueous polysaccharide extract of* Agaricus blazei* Murill on the stimulation of proinflammatory cytokine production in human monocytes and human vein endothelial cells* in vitro*. They reported that* A. blazei* extract is rich in *β*(1,3)-, *β*(1,4)-, and *β*(1,6)-D-glucans (Figure 1) and induces the release of proinflammatory cytokines (IL-1*β*, IL-6, IL-8) and *α*TNF. Another research has also reported that* A. blazei* extract enhances local and systemic inflammation, upregulates proinflammatory molecules, and enhances leukocyte homing to atherosclerosis sites without affecting the lipoprotein profile [50].

Contrary to these results, reports have recently appeared indicating that polysaccharides from* A. blazei* exert anti-inflammatory activities. Song et al. [51] investigated the anti-inflammatory and antiallergic effects of the chloroform-soluble extract of* A. blazei* in mouse bone-marrow-derived mast cells (BMMCs). They found that the extract inhibited the production of IL-6 in phorbol myristate acetate (PMA) plus calcium ionophore A23187-stimulated BMMCs and downregulated the phosphorylation of the serine/threonine kinase AKT. Furthermore, the extract inhibited the degranulation of *β*-hexosaminidase, as well as the production of prostaglandin D(2) and leukotriene C(4). They concluded that their* A. blazei* extract has anti-inflammatory and antiallergic activities, which are regulated by affecting IL-6, prostaglandin D(2), leukotriene C(4), and the phosphorylation of the serine/threonine kinase AKT.

In 2009, Johnson et al. [52] reported a phase I clinical trial in which 15 healthy individuals were given a daily oral dose of 60 mL of aqueous extract of AndoSan (a mushroom extract mixture containing 82%* A. blazei* mycelium, 15%* Hericium erinaceum*, and 3%* Grifola frondosa*) for 12 days. Their results showed a significant* in vivo* reduction in the levels of IL-1*β*, TNF-*α*, IL-6, IL-2, and IL-7 proinflammatory cytokines and unaltered levels of the remaining 12 cytokines. They concluded that the applied extract has an anti-inflammatory effect [52]. In 2011, they conducted another clinical pilot study in order to prove these results. They treated 21 hospitalized patients with inflammatory bowel diseases, ulcerative colitis, and Crohn's disease with the same AndoSan treatment dose (60 mL/d for 12 days). They noticed a significant decrease in plasma levels of the proinflammatory cytokines as well as a decrease in calprotectin, an inflammatory marker, in the faeces of ulcerative colitis patients [53].

Ellertsen and Hetland [54] attributed the apparently contradictory findings reported above to the fact that the effect of* A. blazei* extract differs according to the molecular size of active compound and the type of study performed (i.e.,* in vitro* or* in vivo*). They found that* in vitro* cell culture studies showed increased proinflammatory cytokines, while* in vivo* human studies showed an anti-inflammatory response. They suggested that cells* in vitro* are affected by all substances present in the extract, including high molecular weight *β*-glucans. On the other hand, in human* in vivo* studies, it is mainly the smaller substances which are absorbed into the digestive tract to become active in blood. Additionally, they found that the regulatory genes of the leukocytes affected by the* A. blazei* extract differ in their expression* in vitro* and* in vivo*. Genes regulating proinflammatory cytokines were strongly induced* in vitro*, probably by the high molecular weight *β*-glucans, whereas,* in vivo*, genes controlling anti-inflammatory reactions were upregulated, due to low molecular weight glucans. This hypothesis was supported by the mixed composition of the AndoSan extract, which contained both high and low molecular weight polysaccharides.

Other studies have reported that the protein-bound polysaccharides in the aqueous extract of* A. subrufescens* enhance defence mechanisms against invasive organisms and bacterial infection and reduce the plaque formation of viruses in cell cultures [49, 55, 56].

Additionally, glucans ((1→3)-*β*-D-glucopyranosyl) from* Pleurotus pulmonarius* have been reported to exhibit anti-inflammatory properties [57–59]. These studies demonstrated that the fruiting body, or mycelial extract, of the edible mushroom markedly attenuated or suppressed symptoms associated with dextran sulphate sodium-induced acute colitis in mice. They also reported that, in an* in vitro* study, these glucans inhibited TNF-*α*-dependent activation of NF-*κ*B in human intestinal cells [57]. Furthermore, *β*-glucans from* P. pulmonarius* exhibited an anti-inflammatory response in a model of acute colitis in rats, and when those from* P. ostreatus* were tested, they inhibited leukocyte migration to acetic acid-injured tissues [60, 61]. Recently, Jedinak et al. [62] showed that oyster mushroom concentrate (OMC) containing *α*- and *β*-glucans suppressed LPS-induced dependent activation of TNF-*α*, IL-6, and IL-12 in RAW 264.7 murine macrophage cells, as well as inhibiting LPS-induced production of prostaglandin E2 (PGE2) and nitric oxide (NO). This was mainly attributed to the downregulation of COX-2 and iNOS expression, respectively. They also reported that OMC significantly suppressed LPS-induced production of TNF-*α* in mice and concanavalin A-stimulated proliferation and secretion of INF-*γ*, IL-2, and IL-6 in mouse splenocytes [62].

The methanolic extract of* Caripia montagnei* (mainly polysaccharides of the glucan type) has also been shown to exert anti-inflammatory effects on male Swiss mice and male Wistar rats. The glucans significantly reduced the inflammatory infiltrate produced by thioglycolate-mediated peritonitis by about 75%. Additionally, a significant reduction in the nitric oxide levels was observed in the exudates [63]. Ruthes et al. [64], meanwhile, investigated water and ethanolic extracts of (1→3), (1→6)-*β*-D-glucans from the fruiting bodies of* Lactarius rufus* for their anti-inflammatory effects in both male and female Swiss mice. They found that soluble glucans inhibit inflammatory pain caused by formalin in comparison to the insoluble glucans, concluding that solubility and/or branching degree can alter the activity of *β*-glucans.

Furthermore, the aqueous extract of fucogalactan from* Agaricus bisporus* exhibited anti-inflammatory response in male Swiss mice [65]. It inhibited the neurogenic and inflammatory phases of formalin-induced licking, which they attributed to the decreased iNOS and COX-2 expression. Additionally, heterogalactans isolated by cold aqueous extraction from* Lentinus edodes* have shown anti-inflammatory activities in male Swiss mice [66]. The active fraction was made of fucomannogalactan with a main chain of (1→6)-linked *α*-D-galactopyranosyl units, partially substituted at O-2 with single-unit *β*-D-Man*p* or *α*-L-Fuc*p* side chains. The polysaccharide produced a marked effect against acetic acid induced visceral nociception and inhibited the peritoneal capillary permeability and leukocyte infiltration.

Mushroom polysaccharides with anti-inflammatory properties have been also reported in crude extracts of* Lentinus polychrous*,* Termitomyces albuminosus*, and* Phellinus linteus*. In* L. polychrous*, they inhibited NO and proinflammatory productions by downregulating the gene expressions of proinflammatory mediators, thereby decreasing paw oedema in rats [67]. In* T. albuminosus*, they decreased the acetic acid induced writhing response and the licking time in the late phase in the formalin test in male ICR mice [68]. Additionally, mouse ear swelling was inhibited by 61.8, 79.0, and 81.6% when the animals were treated with the dry matter of the culture broth (1000 mg/kg), crude saponin extract (200 mg/kg), or crude polysaccharide extract (200 mg/kg), respectively. In another study, polysaccharides of* P. linteus* inhibited the mouse ear oedema induced by croton oil, reduced the amount of writhing induced by acetic acid in mice [69], induced heme oxygenase-1 (HO-1) of the RAW 264.7 macrophages, and suppressed iNOS and the subsequent production of nitric oxide by downregulation of iNOS promoter activity in lipopolysaccharide-stimulated macrophages [70].

The polysaccharides of* Pholiota nameko* have been reported to inhibit topical oedema in mouse ears and significantly suppress the development of egg albumin-, carrageenan-, and formaldehyde-induced paw oedema in the animals, and, significantly, did not produce any gastric lesions in rats [71]. The polysaccharides of the golden needle mushroom (*Flammulina velutipes*) are composed of three monosaccharides (glucose, mannose, and xylose) in a molar ratio of 3.5 : 0.8 : 1.4 and have been found to have anti-inflammatory activities and significantly decreased CD4^+^ CD8^+^, ICAM-1, and MPO in both the serum and colon of normal and burned rats [72]. Tibetan mushroom polysaccharides also exhibited an anti-inflammatory activity by significantly inhibiting the formation of granuloma tissue by about 43% as well as significantly decreasing rat paw oedema induced by carrageenan [73]. Polysaccharides extracted from* Cantharellus tubaeformis* [74] and* Lactarius flavidulus* [75] have also been reported to exhibit anti-inflammatory properties.  Table 2 summarizes some of the biological studies carried out with different compounds having anti-inflammatory activities isolated from mushrooms.

### 3.2. Terpenoids

**Figure 2:**
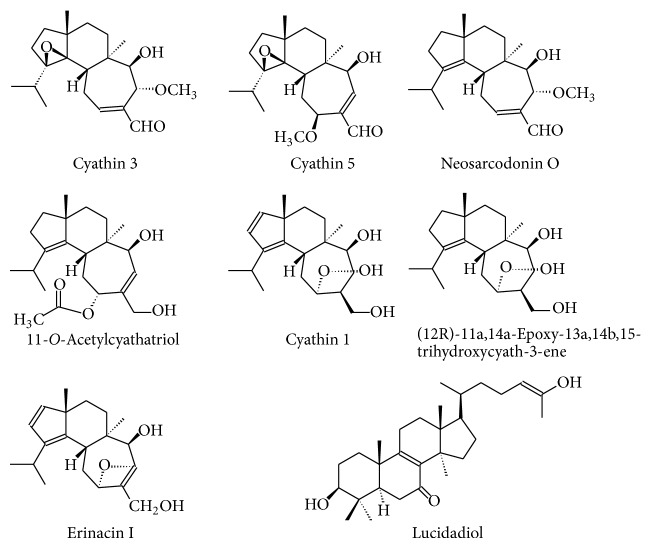
Examples of some terpenoid bioactive compounds isolated from edible mushrooms.

Terpenes are the largest group of anti-inflammatory compounds in mushrooms and have been isolated from a range of different strains (Figure 2). Han et al. [76] isolated five novel cyathane diterpenes, identified as cyathins D-H (1–5), in addition to three well known diterpenes, namely, neosarcodonin, cyathatriol, and 11-O-acetylcyathatriol from the ethylacetate extract of* Cyathus africans*. They found that cyathins D-H 3 and 5, as well as neosarcodonin and 11-O-acetylcyathatriol, showed potent inhibition activity against NO production in lipopolysaccharide-mouse monocyte-activated macrophage RAW 264.7, with IC_50_ values of 2.75, 1.47, 12.0, and 10.73 *μ*M, respectively. Xu et al. [77] isolated three bioactive diterpenes from the ethylactetate extract of* C. hookeri* Berk, namely, cyathin, (12R)-11a,14a-epoxy-13a,14b,15-trihydroxycyath-3-ene, and erinacine I. They also found that each of their three isolated diterpenes showed potent anti-inflammatory activities, inhibiting NO production in mouse monocyte-macrophages RAW 264.7, with an IC_50_ of 15.5, 52.3, and 16.8 *μ*M, respectively.

Another class of terpenoids (triterpenes) have also been shown to exhibit high anti-inflammatory properties [78]. The authors of this study evaluated the anti-inflammatory effects of triterpene extract of* Ganoderma lucidum* in lipopolysaccharide- (LPS-) stimulated macrophages. They reported that the triterpene extract significantly suppressed the secretion of inflammatory cytokine tumour necrosis factor-*α* (TNF-*α*) and interleukin-6 (IL-6), as well as the inflammatory mediators nitric oxide (NO) and prostaglandin E2 (PGE2), from LPS-stimulated murine RAW 264.7 cells. Additionally, the LPS-dependent expression of inducible nitric oxide synthase (iNOS) and cyclooxygenase 2 (COX-2) in RAW 264.7 cells were downregulated. The anti-inflammatory effects of the triterpene extract have been attributed to the inhibition of transcription factor NF-*κ*B, as observed in the decreased NF-*κ*B-DNA binding activity, as well as the suppression of p65 phosphorylation in the treated LPS-stimulated macrophages. Additionally, the extract inhibited LPS-dependent AP-1-DNA binding activity and downregulated the expression of AP-1 subunit c-Jun. Furthermore, the activity of MAP kinases has been suppressed, as observed by the downregulation of LPS-induced phosphorylation of ERK1/2 and JNK.

**Figure 3:**
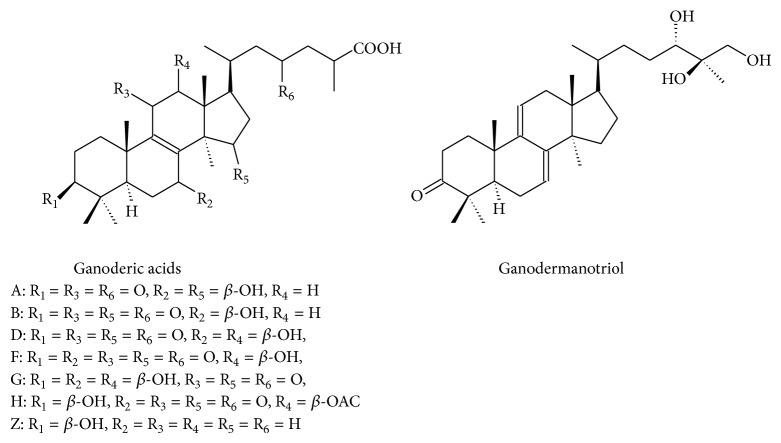
Structures of different ganoderic acids and derivative isolated from* G. lucidum*.

Furthermore, different lanostane-type triterpenic acids with potent anti-inflammatory properties have been isolated from* G. lucidum* [79, 80]. Akihisa et al. [81] reported that nine lucidenic acids and four ganoderic acids (Figure 3), isolated from the fruiting bodies of* G. lucidum*, significantly inhibited 12-*O*-tetradecanoylphorbol-13-acetate induced inflammation (1 *μ*g/ear) in mice, with IC_50_ values between 0.07 and 0.39 mg/ear.

**Figure 4:**
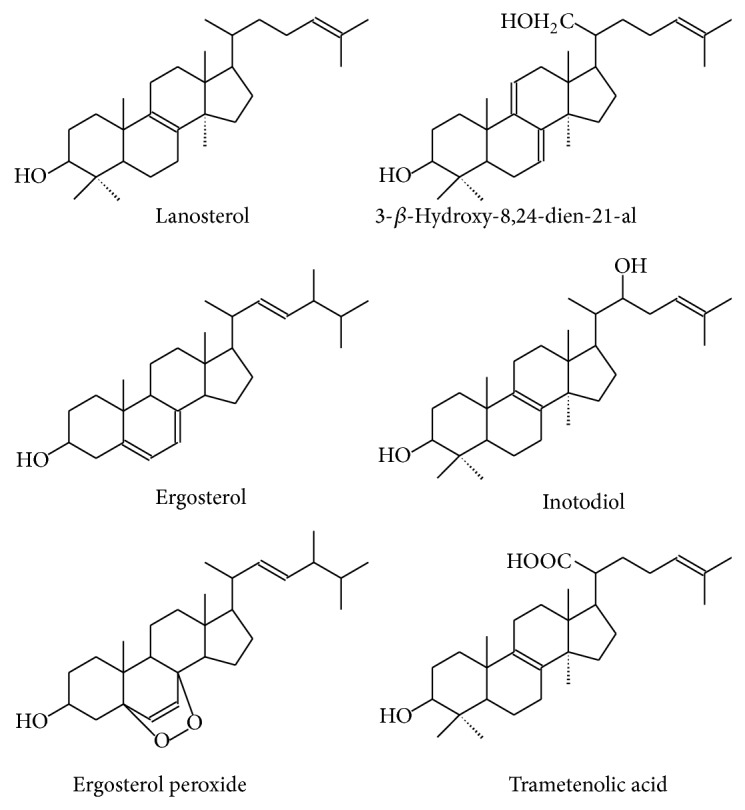
Structures of different sterol compounds isolated from* I. obliquus*.

Different sterols (Figure 4) with potent anti-inflammatory activities have been also isolated and purified from the edible mushroom* Inonotus obliquus*. These significantly inhibited the levels of IL-1*β*, IL-6, and TNF*α* in a dose-dependent manner in murine macrophage RAW 164.7 cells [82]. Additionally, Park et al. [83] found that the methanolic extract of* I. obliquus* significantly inhibited NO production, prostaglandin E_2_, and TNF-*α* in LPS-stimulated RAW 264.7 macrophages. Ma et al. [84] isolated and identified six known triterpenes from the sclerotia of* I. obliquus*. They reported that trametenolic acid, ergosterol peroxide, 3*β*-hydroxy-8,24-dien-21-al, ergosterol, and inotodiol inhibited NO production and NF-*κ*B luciferase activity in macrophage RAW 264.7 cells, with an inhibition percentage of 50.04, 36.88, 20.36, 6.00, and 3.13%, respectively.

### 3.3. Peptides

In addition to the previous bioactive compounds, anti-inflammatory peptides of different molecular weights have been isolated from mushrooms. Cordymin, a low molecular weight peptide (10,906 Da), has been purified from the medicinal mushroom* Cordyceps sinensis* [85, 86] and from* C. militaris* [87]. This peptide significantly inhibited the infiltration of polymorphonuclear cells and IR-induced upregulation of C3 protein produced in the brain, interleukin-1*β*, and tumour necrosis factor-*α*, which had a neuroprotective effect on the ischemic brain, due to the inhibition of inflammation. Agrocybin represents another peptide isolated from the edible mushroom* Agrocybe cylindracea*. Agrocybin exhibited antifungal activity against* Mycosphaerella arachidicola*, with an IC_50_ value of 125 *μ*M at different temperatures up to 80°C [88].

### 3.4. Phenolics

**Figure 5:**
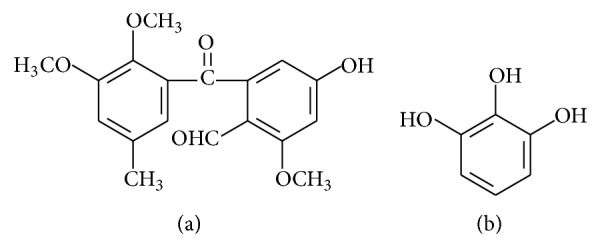
Chemical structures of (a) daldinal and (b) pyrogallol.

Many edible mushrooms have been found to exhibit anti-inflammatory properties due to the presence of some phenolic compounds (Figure 5). For example, the common phenolic molecule pyrogallol has been extracted from* Agaricus bisporus*,* Cantharellus cibarius*, and* Lactarius deliciosus* [89, 90]. Pyrogallol inhibited NO production and iNOS, IL-1*β*, and IL6 mRNAs expression in response to LPS-stimulated RAW 364.7 macrophages [91]. Additionally, daldinals have been purified from* Daldinia childiae*. They strongly suppressed the LPS-induced production of NO in RAW 264.7 cells, with IC_50_ values ranging between 4.6 and 15.2 *μ*M depending on the derivative [92]. This effect was attributed to the inhibition of iNOS mRNA synthesis.

**Figure 6:**
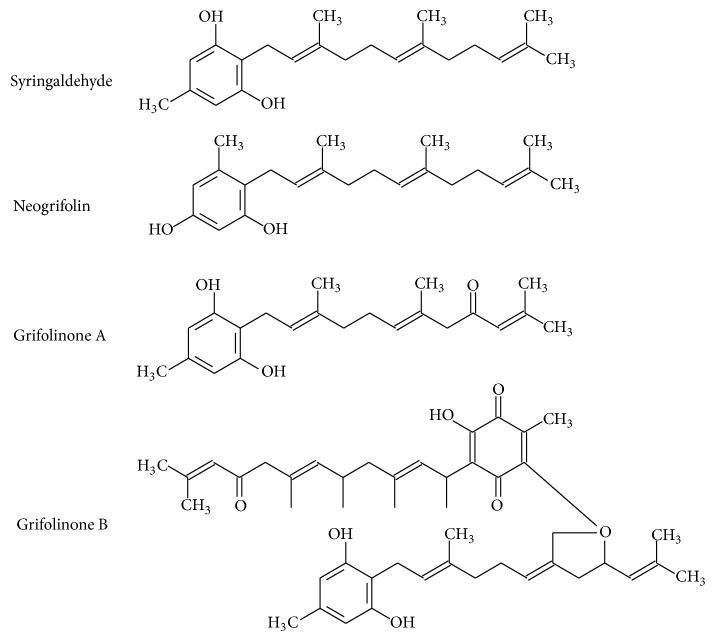
Grifolin and neogrifolins isolated from* Albatrellus* mushrooms.

Grifolin and grifolin derivatives (Figure 6) represent another class of farnesyl phenolic compounds which have been isolated from the edible mushroom* Albatrellus ovinus* and which exhibit anti-inflammatory properties [93]. Grifolins showed significant inhibition of NO production stimulated by LPS in RAW 264.7 cells, with IC_50_ values ranging between 22.9 and 29 *μ*M [94], as well as inhibiting histamine release from rat peritoneal mast cells [95].

### 3.5. Miscellaneous

**Figure 7:**
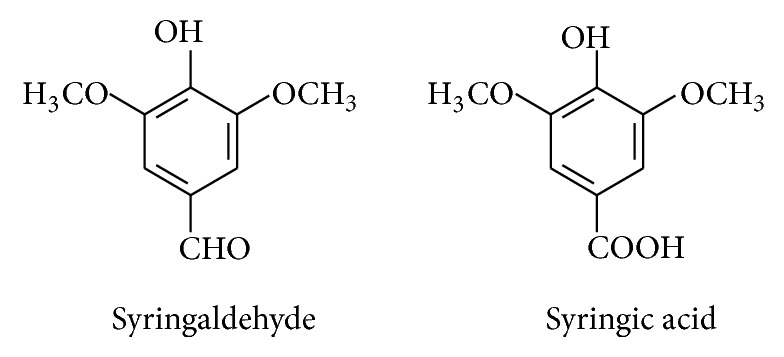
Structure of anti-inflammatory compounds isolated from edible truffles.

The ectomycorrhizal edible truffle* Elaphomyces granulatus* has been evaluated for its anti-inflammatory effects. The 95% ethanolic extract of the fruiting bodies contained two active aromatic compounds with anti-inflammatory and antioxidant activities, namely, syringaldehyde and syringic acid (Figure 7). The anti-inflammatory properties of these two low molecular weight organic compounds were proven by their inhibition of the mediator cyclooxygenase-2 (COX-2) enzyme in mouse macrophage (RAW 264.7). The crude ethanolic extract caused about 68% inhibition of the enzyme activity at a concentration of 50 *μ*g/mL. The inhibitory effect of the purified syringaldehyde and syringic acid on COX-2 activity showed a dose-dependent effect, with an IC_50_ of 3.5 and 0.4 *μ*g/mL, respectively, [19, 96].

Agaricoglycerides represent a new class of fungal secondary metabolites that constitute esters of chlorinated 4-hydroxy benzoic acid and glycerol and are produced in the culture of different edible mushrooms such as* Grifola frondosa* and* Agaricus macrosporus*. They also exhibited significant anti-inflammatory activity. The agaricoglycerides isolated from* Grifola frondosa* (AGF) showed potent anti-inflammatory activity since they were able to reduce the level of many mediators, such as IL-1*β*, NF-*κ*B, ICAM-1, COX-2, and iNOS in animal models [97]. This study showed that an oral dose of 500 mg/kg/day of the ethanolic extract of an agaricoglyceride mixture showed strong anti-inflammatory activity in a Wister rat animal model.

## 4. Future Perspectives

This review has demonstrated the importance of mushrooms as potential biofactories for the production of natural anti-inflammatory metabolites of highly diversified chemical structure. The bioactivities of these compounds are exhibited through the downregulation of different types of inflammatory mediators. In addition to the high potential application of anti-inflammatory metabolites from mushrooms in forms of unpurified extract and extra pure compounds in medical applications, they can also be used in cosmeceutical products as safe and natural active ingredients without undesired side effects. However, the future medical application of anti-inflammatory compounds isolated from mushrooms faces five main challenges. Firstly, most of the studied mushrooms are not cultivable in greenhouses, and thus their availability is both seasonal and highly affected by changes in the weather. Secondly, the contents of the bioactive ingredients vary widely between samples, dependent on the collection time and procedure, the season, and the environment. Thirdly, mushroom cultivation in greenhouses is an open system and is not run according to the current Good Manufacturing Practice (cGMP) requirements for the production of bioactive medicinal compounds. Therefore, more research should be done on the development of mushroom cultivation processes in submerged cultures under fully sterile conditions so as to produce bioactive metabolites for pharmaceutical applications. Fourthly, in most of the studies conducted so far, the anti-inflammatory activities of mushrooms were demonstrated using crude mushroom extracts or solvent extracts of different metabolites in a mixed form. It is necessary, therefore, to isolate and identify the active metabolites for a better understanding of the anti-inflammatory properties of each particular compound and the possible side effects, if any. This step is necessary to upgrade the crude extract from a nutraceutical to pharmaceutical market after proper product formulation and clinical trials to determine the proper dose and claims. Fifthly, there is a lack of validated standard testing protocols to guarantee the quality and the efficacy of mushroom products for pharmaceutical applications. Overall, therefore, more research is required to overcome the challenges mentioned above before macrofungi can be fully accepted as one of the major biofactories for the production of anti-inflammatory medicine.

## Figures and Tables

**Table 1:** Anti-inflammatory compounds of mushrooms.

Mushroom species	Biomaterial source	Extracting solvent	Bioactive compound	References
*Agaricus blazei *	WM	1	Polysaccharides	[51]
*A. bisporus *	WM	3	Pyrogallol, hydroxybenzoic acid derivatives, flavonoids.	[91]
	FB	4	Polysaccharide (fucogalactan)	[65]
*A. subrufescens *	WM	2	Polysaccharide (proteoglucan)	[55]
*Agrocybe aegerita *	FB	3	Fatty acids	[88]
*A. cylindracea *	FB	2	Agrocybin	[98]
*Albatrellus caeruleoporus *	FB	3	Phenolic compound (Grifolinones A, B)	[93, 94]
*Amanita muscaria *	FB	2, 3, 4	Crude extract	[99]
*Boletus edulis *	WM	3	polysaccharides,	[91]
*Cantharellus cibarius *	WM	3	Pyrogallol, flavonoids, polysaccharides	[91]
*C. tubaeformis *	WM	2	Polysaccharides	[74]
*Cordyceps militaris *	SC/FB	4	Crude extract	[100]
*C. pruinosa *	FB	3	Crude extract	[101]
*Caripia montagnei *	FB	3	Polysaccharides (Glucans)	[63]
*Cyathus africanus *	SC	5	Diterpenoid	[76]
*C. hookeri *	SC	5	Diterpenoid	[77]
*Daldinia childiae *	FB	N.M.	Benzophenones (daldinals A-C)	[92]
*Elaphomyces granulatus *	FB	4	Syringaldehyde, syringic acid	[19, 96]
*Flammulina velutipes *	WM	4	Polysaccharides	[72]
*Fomitopsis pinicola *	SC	4	Polysaccharides	[102]
*Grifola frondosa *	SC	5, 6	Agaricoglycerides	[97]
	SC	3, 5,	Ergosterol (1-oleoyl-2-linoleoyl-3-palmitoylglycerol)	[103]
*Ganoderma lucidum *	FB	4	Triterpene	[78]
	FB	4	Lucidenic acid, ganoderic acid	[81]
*Geastrum saccatum *	FB	4	Polysaccharides (*β*-glucans)	[104]
	FB	2, 4, 5	Crude extract	[82]
*Inonotus obliquus *	FB	8, 5	Ergosterols, lanosterol, inotodiol, trametenolic acid	[84]
	FB	3	Crude extract	[83]
	FB	4	Crude extract	[105]
*Lactarius deliciosus *	WM	3	Pyrogallol, flavonoids	[91]
*L. rufus *	FB	2, 4	Polysaccharides: (1→3), (1→6)-*β*-D-glucans	[64]
*Lentinus edodes *	FB	2	Heterogalactan (fucomannogalactan) with main chain of (1→6)-linked *α*-D-glactopyranosyl unit	[66]
*L. polychrous *	SC	4	Crude extract	[67]
*Lyophyllum decastes *	FB	3	Polysaccharides: (1–3) and (1–6) *β*-D-glucans	[106]
*Phellinus linteus *	FB	9	Polysaccharides (proteoglycan)	[69]
	FB	4, 7, 9	Crude extract	[107]
*Pholiota nameko *	WM	4, 6, 10	Polysaccharides	[71]
*Pleurotus pulmonarius *	FB	N.M.	Polysaccharides (1→3), (1→6)-linked *β*-glucan	[60]
	FB	2, 4	Polysaccharides (Glucan)	[57]
*Poria cocos *	SC	4	Polysaccharides	[108]
*Termitomyces albuminosus *	SC/FB	4	Crude polysaccharide extract	[68]

WM, whole mushroom; SC, submerged culture; FB, fruiting bodies.

Solvent: N.M., not mentioned; 1, chloroform; 2, water; 3, methanol; 4, ethanol; 5, ethyl acetate; 6, acetone; 7, n-hexane; 8, petroleum ether; 9, n-butanol; 10, acetyl ether.

**Table 2:** Examples of biological studies performed with anti-inflammatory compounds from mushrooms.

Bioactive compound/mushroom species	Assay model	Results/mechanism of action	References
Polysaccharides			
* Agaricus blazei *	(i) Mouse bone marrow-derived mast cells (BMMCs) stimulated with PMA + A23187	(i) Inhibition of IL-6 production, downregulation of phosphorylation of Akt, inhibition of *β*-hexosaminidase degranulation, inhibition of prostaglandin D(2), and leukotriene C(4) production.	[51]
	(i) Male Swiss mice (acetic acid induced inflammation)	(i) Dose-dependent anti-inflammatory response, inhibition of leukocyte migration (82%), IC_50_ of 1.19 (0.74–1.92) mg/kg, 3 mg/kg i.p. glucan injection reduced 85% of writhes	[60]
* Pleurotus pulmonarius *	(ii) Mice (3.5% dextran sulfate sodium, DSS in drinking water for 14 days, with 20 mg fruiting body or mycelia extract/mouse/day)	(ii) Fruiting body and mycelia extracts suppressed inflammatory reactions *in vivo* in DSS induced colonic inflammation by downregulating TNF-*α* secretion and inhibiting NF-*κ*B activation	[57, 58]
	(iii) Acetic acid induced colitis in rats (2% pleuran, or 0.44% hydrogel for 4 weeks)	(iii) Reduction in macroscopic damage score by 51 and 67% for pleuran diet and hydrogel, respectively; reduction in the activity of myeloperoxidase and neutrophil infiltration	[61]
	(iv) Murine macrophage RAW264.7 cells, female Balb/C mice	(iv) Suppression of LPS-induced dependent activation of TNF-*α*, IL-6, and IL-12, inhibition of LPS-induced production of PGE2 and NO. Suppression of LPS-induced production of TNF-*α* in mice and concanavalin A-stimulated proliferation and secretion of INF-*γ*, IL-2, and IL-6 in mouse splenocytes	[62]
* Caripia montagnei *	(i) Male Swiss mice treated with 10, 30, and 50 mg/kg with mushroom glucan	(i) 50 mg/kg glucan reduced inflammatory infiltrate produced by thioglycolate-induced peritonitis by 75.5%, reduced NO level, IL-1ra, IL-10, and IFN-*γ*	[63]
* Lactarius rufus *	(i) Swiss mice, formalin-induced nociception, 30 mg/kg i.p. of fruiting body extract (soluble, insoluble, and modified)	(i) Inhibition of neurogenic pain by 36, 47, and 58% for soluble, insoluble, and modified glucans, respectively	[64]
* A. bisporus *	(i) Male Swiss mice, formalin-induced licking	(i) Inhibition of neurogenic and inflammatory phases, antinociceptive effect with IC_50_ of 36.0 (25.8–50.3 mg/kg) (ii) Decreased iNOS and COX2	[65]
* Lentinus edodes *	(i) Male Swiss mice, acetic acid induced inflammation, 3–100 mg/kg i.p. fruiting body concentrate	(i) Inhibition of induced nociception with IC_50_ of 13.8 (7.8–23.5) mg/kg, 97% inhibition at 100 mg/kg (ii) Inhibition of peritoneal capillary permeability and leukocyte infiltration (76% inhibition), IC_50_ 13.9, 8.2–23.7, and 100% inhibition, IC_50_ 6.5, 1.5–28.2 mg/kg, respectively	[66]
* L. polychrous *	(i) Carrageenan-induced paw edema in male Sprague-Dawley rats, murine macrophage RAW 264.7 cells	(i) Dose-dependent inhibition of NO, intracellular O_2_^−^ production (ii) Decreased expression of iNOS, IL-1*β*, IL-6, TNF-*α*, and COX-2	[67]
* Termitomyces albuminosus *	(i) Acetic acid induced writhing in male ICR mice, formalin test, xylene, and carrageenan induced ear edema	(i) Inhibition of ear swelling by 61.8, 79.0, and 81.6% for treatment with dry matter of the culture broth (1000 mg/kg), crude saponin extract (200 mg/kg), or crude polysaccharide extract (200 mg/kg), respectively	[68]
* Phellinus linteus *	(i) Croton oil induced ear edema and acetic acid induced writhing in male ICR mice	(i) Extract treatment with 1 mg/ear gave 45 and 41.5% inhibition in ear plug weight and thickness, respectively, oral administration of extract (100–400 mg/kg) inhibited writhing number (35.9–68.9%)	[69]
* Pholiota nameko *	(i) Xylene induced ear edema, adult Swiss mice and Sprague-Dawley rats, formaldehyde, egg albumin, and carrageenan induced paw edema in rats and mice	(i) Extract (5 mg/ear) inhibited ear edema, suppression of egg albumin, carrageenan and formaldehyde-induced paw edema at 100–400 mg/kg i.p., 10.96–43.75% inhibition of granuloma tissue growth, no production of gastric lesions in rats	[71]
* Flammulina velutipes *	(i) Male Wistar rats, fed 100–300 mg/kg mushroom for 30 days	(i) Decreased levels of CD4^+^ CD8^+^, MPO, and ICAM-1, with increased level in IL-10 in serum	[72]

Terpenoids			
* Cyathus africanus *	(i) Mouse monocyte-macrophage RAW 264.7 cells, NO assay	(i) Cyathins D-H 3 and 5, neosarcodonin, and 11-O-acetylcyatha-triol inhibited NO production with an IC_50_ value of 2.75, 1.47, 12.0, and 10.73 *μ*M, respectively	[76]
* C. hookeri *	(i) Mouse monocyte-macrophage RAW 264.7 cells, NO assay	(i) Inhibition of NO production with an IC_50_ of 15.5, 52.3, and 16.8 *μ*M, respectively.	[77]
*Ganoderma lucidum *	(i) LPS-stimulated murine macrophage RAW 264.7 cells, NO assay	(i) Inhibition of TNF-*α*, IL-6, NO, and PGE2, downregulation of iNOS and COX-2, inhibition of NF-*κ*B, decreased NF-*κ*B-DNA binding activity, and suppression of p65 phosphorylation	[78]
	(ii) Acetic acid induced ear edema in female ICR and SENCAR mice	(ii) Significant inhibition of inflammation (1 *μ*g/ear) in mice with IC_50_ values between 0.07 and 0.39 mg/ear, with inhibition ratio ranging from 58 to 97%	[81]
		(i) Reduced nitrate levels by an average of 50%, dose-dependent inhibition of IL-1*β*, IL-6, and TNF*α*	[82]
* Inonotus obliquus *	(i) Murine macrophage RAW 164.7 cells	(ii) Trametenolic acid, ergosterol peroxide, 3*β*-hydroxy-8,24-dien-21-al, ergosterol and inotodiol inhibited NO production, and NF-*κ*B luciferase activity, with an inhibition percentage of 50.04, 36.88, 20.36, 6.00, and 3.13%, respectively	[84]
		(iii) Methanolic extract inhibited production of NO, prostaglandin E_2_, and TNF-*α*, inhibition of mRNA expression of iNOS and COX-2	[83]

Peptides			
* Cordyceps sinensis *	(i) Acetic acid induced inflammation in mice	(i) Decreased level of TNF-*α*, IL-1*β*, dose-dependent inhibition of abdominal constrictions	[86]

Phenolics			
* Lactarius deliciosus *	(i) LPS-stimulated RAW 364.7 macrophage cells, nitrite, and cytokine assays	(i) 0.5 mg/mL mushroom extract inhibited NO production and expression of iNOS, IL-1*β*, and IL6 mRNAs	[91]
* Daldinia childiae *	(i) LPS-stimulated RAW 264.7 macrophage cells	(i) Daldinals suppressed NO production with IC_50_ values ranging between 4.6 and 15.2 *μ*M and inhibited iNOS mRNA synthesis	[72]
* Albatrellus caeruleoporus *	(i) LPS-stimulated mouse macrophage RAW 264.7 cells	(i) Grifolins inhibited NO production with IC_50_ values ranging between 22.9 and 29 *μ*M	[94]

Syringaldehyde and syringic acid			
* Elaphomyces granulates *	(i) Mouse macrophage RAW 264.7 cells	(i) Crude ethanolic extract (50 *μ*g/mL) inhibited COX-2 activity by 68%, purified syringaldehyde, and syringic acid inhibited COX-2 activity in a dose-dependent manner, with an IC_50_ of 3.5 and 0.4 *μ*g/mL, respectively	[96]

Agaricoglycerides			
* Grifola frondosa *	(i) Acetic acid- and formalin-induced inflammation in Wister rats, treatment with orally fed extracts (100–500 mg/kg/day)	(i) 500 mg/kg/day inhibited induced upregulation of NF-*κ*B and the production of IL-1*β*, TNF-*α*, ICAM-1, COX-2, and iNOS, suppressed acetic acid induced abdominal constrictions and formalin-induced spontaneous nociceptive behavior	[97]
